# Joint Manifestations in Inflammatory Bowel Diseases, “Red Flags” for the Early Recognition and Management of Related Arthropathies: A Narrative Review

**DOI:** 10.3390/jcm14051558

**Published:** 2025-02-26

**Authors:** Ilenia Di Cola, Luca Vallocchia, Paola Cipriani, Piero Ruscitti

**Affiliations:** Department of Biotechnological and Applied Clinical Sciences, University of L’Aquila, 67100 L’Aquila, Italy; luca.vallocchia@graduate.univaq.it (L.V.); paola.cipriani@univaq.it (P.C.); piero.ruscitti@univaq.it (P.R.)

**Keywords:** inflammatory bowel diseases, spondyloarthritis, red flags

## Abstract

Inflammatory bowel diseases (IBDs), including Crohn’s disease and ulcerative colitis, frequently present with extra-intestinal manifestations. Virtually all patients with IBD could be at risk for developing inflammatory arthropathies within the spectrum of spondyloarthritis (SpA). In this context, prompt recognition of musculoskeletal “red flags” (lower back pain, dactylitis, enthesitis, swelling of peripheral joints, musculoskeletal chest pain, family history of SpA, psoriasis, and anterior uveitis) is crucial for early referral and multidisciplinary management by gastroenterologists and rheumatologists. Recent advances have refined diagnostic tools including questionnaires, alongside imaging modalities and laboratory markers, enhancing the detection of SpA in IBD patients. Effective treatment strategies targeting both gastrointestinal and musculoskeletal symptoms may significantly reduce long-term morbidity in these patients. In this narrative review, we aimed to underscore the importance of integrating clinical, diagnostic, and therapeutic approaches for optimal patient management and outcome over time.

## 1. Introduction

Historically, a link between the gut and arthritis has been proposed, identifying the occurrence of arthritis as a further extra-intestinal manifestation of patients with inflammatory bowel disease (IBD) [1]. The latter affects almost 3.2 million individuals in Europe, mainly due to ulcerative colitis (UC) and Crohn’s disease (CD), but also undifferentiated colitis or microscopic colitis may be recognized [2]. CD is characterized by focal intestinal inflammation and discontinuous “skip lesions”, with varying degrees of inflammation, which are adjacent to areas of normal-appearing mucosa, unlike the continuous inflammation in UC observed in the colon and rectum [3]. The intestinal symptoms may be various, from mild to severe form, including diarrhea often with blood or pus, intestinal bleeding, abdominal pain, weight loss, and fatigue [4]. In parallel with intestinal features, patients with IBD may display extra-intestinal manifestations, including inflammatory joint diseases [5]. Articular manifestations usually exacerbate during colitis flare-ups and reduce during the remission of gut symptoms [6]. Although pathognomonic signs of joint manifestations may not be present in the early stages, virtually all patients with IBD may be at risk of developing inflammatory arthropathies within the spectrum of spondyloarthritis (SpA) [7]. The latter prompted further research into the connection between the development of arthritis along with the phases of bowel inflammation [8]. These observations have initially led to the introduction of the term “enteropathic arthritis”, an umbrella of conditions that consider any type of arthritis associated with intestinal inflammatory disease [9]. Subsequently, the code denoting “enteropathic arthropathies” has been modified into IBD-related arthropathies. Clinically, IBD-related arthropathies may be categorized in two main forms: i. Type I arthropathy is characterized by oligoarthritis involving large joints and occurring together with IBD flare-ups; ii. Type II arthropathy is identified by persistent arthritis in both small and large joints, presenting independently of IBD activity [10]. In addition, these patients with IBD, independently codified as Type I or Type II arthropathy, may be characterized by the involvement of the spine, manifesting with inflammatory back pain (IBP) as part of the SpA spectrum. In addition, other possible joint signs due to IBD-related arthropathies may include the presence of enthesitis, dactylitis, and hypertrophic osteoarthropathy [11]. Several studies report the prevalence of enthesitis in adult patients with IBD, ranging from 7% to 50% [12,13,14]. The prevalence of SpA manifestations in IBD patients has been estimated to range from 17% to 45.7% [15]. Recently, in a Norwegian cohort of IBD patients (the IBSEN cohort), 36% met the Assessment in Spondyloarthritis international Society (ASAS) criteria for SpA, with 28% having peripheral features and 8% having axial manifestations [16]. Furthermore, a meta-analysis showed that about 13% of patients with IBD may experience the two types of inflammatory peripheral arthritis, whereas 10% may experience sacroiliitis. Moreover, 3% of patients with IBD may meet the classification criteria for ankylosing spondylitis (AS) [11,12,14]. Furthermore, the increases in IBD incidence, which has been observed in young patients over the last three decades, may lead to an increased development of IBD-related arthropathies, thus suggesting the importance of an accurate evaluation of musculoskeletal signs and symptoms [17]. In fact, the occurrence of arthritis in patients with IBD may lead to additional considerations in their management from disease activity control to treatment. This highlights the need for a multidisciplinary approach involving not only gastroenterologists but also rheumatologists.

In this work, we aimed to describe the signs and symptoms of IBD that may be considered “red flags” for further investigating the possible musculoskeletal involvement in IBD patients.

## 2. Typical Clinical Picture of Patients with IBD-SpA

**Figure 1:**
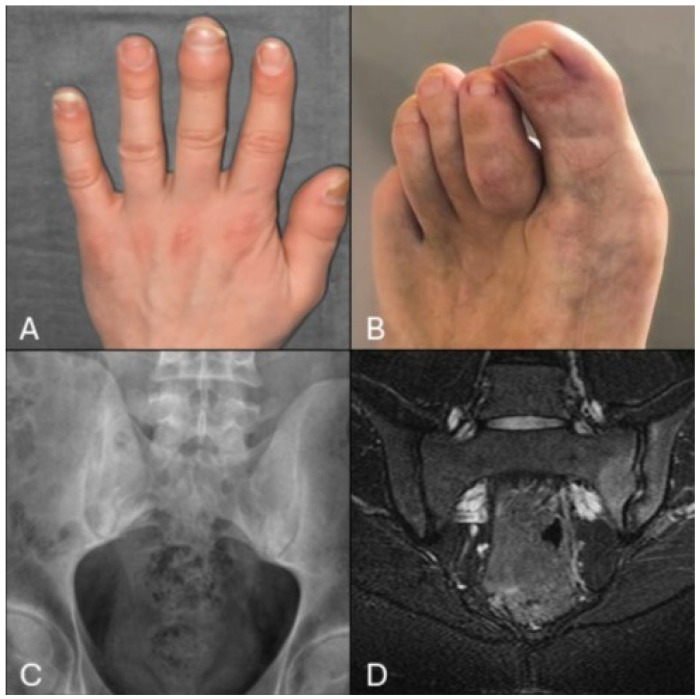
Typical clinical picture of patients with IBD-SpA: (**A**) distal interphalangeal arthritis of the 3rd finger; (**B**) dactylitis of the 2nd toe, often referred to as “sausage digit”; (**C**) plain radiograph of the pelvis showing sacroiliac joints sclerosis; (**D**) MRI of the pelvis revealing intraspongious edema of the left sacroiliac joint, a key feature of axSpA.

The clinical presentation of IBD-SpA is highly heterogeneous, encompassing a wide range of symptoms and manifestations that may significantly vary between patients, including both axial and peripheral involvement, psoriasis, and uveitis. Patients with IBD-SpA usually first report inflammatory musculoskeletal pain, particularly lower back pain and arthralgias in large joints, to their family doctor and/or gastroenterologist. Usually, when patients with IBD come to the rheumatologist for inflammatory joint pain, they have already tried various approaches, including lifestyle modifications and over-the-counter medications. However, these treatments are insufficient to stop the progression of latent SpA and may provide only partial relief of symptoms. If unequivocal signs are not considered, paucisymptomatic patients may continue with this approach for a prolonged period, thus increasing the diagnostic delay and the risk of long-term musculoskeletal complications, including persistence of chronic pain and loss of joint function. Therefore, it is important to identify and address the initial symptoms early (Figure 1).

## 3. Clinical Connection Between IBD and Inflammatory Rheumatic Manifestations

IBD-related musculoskeletal involvement may manifest in various clinical forms in the context of the SpA spectrum. This term refers to the involvement of both axial (axSpA) and peripheral (pSpA) joints, with the primary feature being the inflammation of both the synovium and the enthesis (the attachment sites of tendons and ligaments to bone) [18]. SpA is a potentially disabling inflammatory arthritis of the spine, typically presenting as chronic back pain, with onset usually before the age of 45 years [19]. In the early 1990s, the introduction of the Amor Criteria [20] and the European Spondyloarthropathy Study Group (ESSG) Criteria [21] allowed researchers to classify patients with IBD and musculoskeletal symptoms as having SpA for the first time. The ASAS criteria introduced in 2009 provided further refinement for the classification of SpA, extending their applicability to IBD-related SpA (IBD-SpA) [22]. In IBD patients, musculoskeletal symptoms may appear before the onset of gastrointestinal issues, occur simultaneously, or emerge during IBD flare-ups [21]. These overlapping symptoms may complicate diagnosis, but their early recognition is crucial to improve long-term outcomes and minimize patient disability [23].

## 4. Red Flags

**Figure 2:**
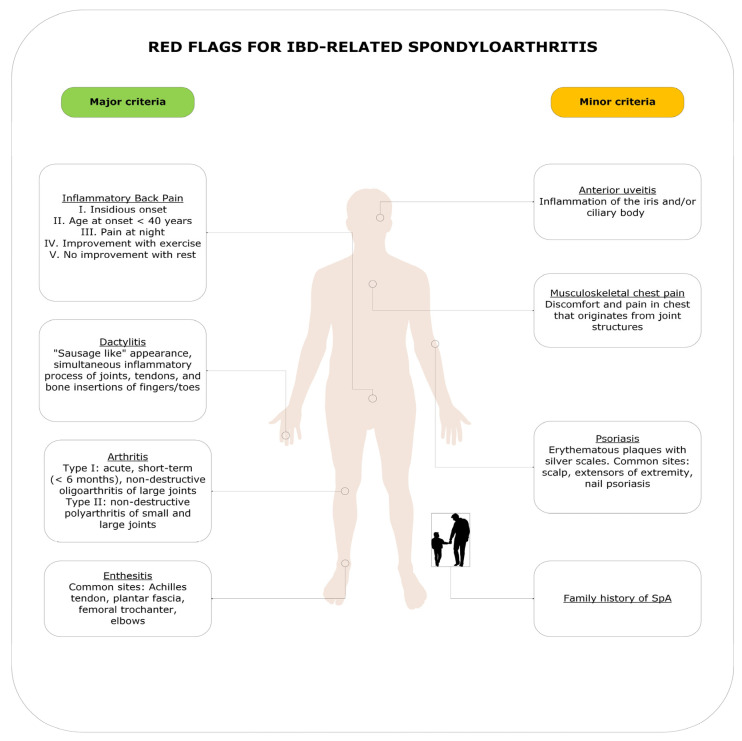
Red flags that suggest the possible early musculoskeletal involvement in IBD patients. Rheumatologic referrals should be considered when at least one major criterion or three minor criteria are present.

The term “red flags” refers to the early signs and symptoms that suggest possible musculoskeletal involvement in IBD patients, thus requiring a rapid referral to a rheumatologist for further evaluation and management [23]. These clinical indicators have been developed by both rheumatologists and gastroenterologists and are based on data collected through various questionnaires and screening tools [24]. In 2018, a systematic review proposed a set of major red flags for a prompt referral to a rheumatologist [25]. The review identified four major criteria and four minor criteria. The major criteria include i. history of chronic lower back pain; ii. dactylitis; iii. enthesitis; and iv. pain/swelling of peripheral joints. The minor criteria include i. musculoskeletal chest pain; ii. family history of SpA; iii. psoriasis; and iv. anterior uveitis [26]. Even though there is no consensus on the significance of each criterion, a rheumatologic referral should be considered when at least one major criterion or three minor criteria are present [25] (Figure 2).

### 4.1. Major Criteria

#### 4.1.1. Inflammatory Back Pain and Sacroiliitis

One of the most prominent red flags for musculoskeletal involvement in IBD is the presence of IBP, which is defined as chronic pain localized in the axial spine and sacroiliac joints [27]. According to ASAS criteria, IBP is typically codified by five clinical items: i. insidious onset; ii. age at onset less than 40 years; iii. pain at night; iv. improvement with exercise; and v. no improvement with rest [28]. These criteria are useful in clinical practice since they may suggest an early identification of inflammatory involvement of the spine. In the context of IBD, IBP is usually characterized by a gradual onset of pain, which typically worsens over weeks or months, rather than the acute, sudden pain, which is observed with mechanical injuries [29]. In addition, morning stiffness may be described as usually lasting more than 30 min and improving with movement [28]. These patients may also report back pain at night, which may impair normal sleep, and it is often relieved by changing positions or getting up [30]. Diagnostic evidence of IBP is often indicated by the presence of sacroiliitis, which occurs in 4% to 30% of IBD patients [14]. In addition, the positive clinical response to NSAIDs could be considered as an additional feature to identify IBP in IBDs [28]. IBP is one of the most helpful clues in diagnosing IBD-SpA. Thus, a prompt identification of IBP is crucial for the appropriate management of these patients.

#### 4.1.2. Arthritis

Arthritis is characterized by acute or chronic inflammation of a joint [31], presenting as a painful, swollen, red, and warm joint with significant functional limitation, although this clinical picture may be subtle on physical examination [14]. The presence of peripheral arthritis in CD and UC is estimated to be 10–20% and 4–14%, respectively, but it is not necessarily associated with latent SpA. Thus, a clinical and diagnostic evaluation to differentiate peripheral arthritis from other causes of articular or extra-articular pain is required [32]. Patients with CD-related sacroiliitis are more likely to develop peripheral arthritis, especially those patients who develop perianal or upper gastrointestinal involvement [33]. While peripheral arthritis may be the only articular manifestation, sacroiliitis in some patients may occur later. A recent study of 361 IBD patients with a history of peripheral arthritis has revealed a 41% prevalence of undiagnosed sacroiliitis on radiographic imaging [34]. Concerning the “classic” peripheral involvement in IBD, Type I arthropathy is an acute, short-term (less than 6 months), non-destructive oligoarthritis, which typically affects the large joints of the lower limbs like the knees. This type of arthritis often appears early or even before bowel symptoms, and it is usually linked with IBD flare-ups [10]. Type II arthropathy is recognized as a non-destructive polyarthritis, which may affect both small and large joints. This kind of joint involvement, if not treated, may last for months, with frequent relapses independent of IBD activity [10].

#### 4.1.3. Enthesitis

Enthesis is the anatomical insertion site of a force-generating unit to bone; an enthesopathy is defined as any pathological change affecting an enthesis [35]. The most common sites of enthesopathies include the Achilles tendon, plantar fascia, femoral trochanter, or elbows [36]. Clinically, the inflammation in enthesitis may result in swelling and pain at the affected site, often exacerbated by movement or pressure [18]. Over time, chronic inflammation at these sites may lead to bone erosions, enthesophytes (bone spurs), or even fibrosis and calcification of the tendon or ligament attachments. Interestingly, the prevalence of enthesitis has been reported to increase among IBD patients receiving therapy with an anti-α4β7 integrin monoclonal antibody (vedolizumab) [37]. This distinct entity, termed ‘vedolizumab-associated enthesitis’, cannot be classified as an extra-intestinal manifestation of IBD [38].

#### 4.1.4. Dactylitis

Another clinical sign to be considered in the presence of IBD-SpA is dactylitis, or the so-called “sausage digit”. It describes swelling of fingers or toes due to a simultaneous inflammatory process of the joints, tendons, and bone insertions [39]. Thus, unlike typical joint inflammation, which may be confined to a specific joint, dactylitis affects the entire finger or toe, giving it a characteristic “sausage-like” appearance. The swelling is often uniform, extending from the base of the digit to the tip [40]. The affected digit may be painful, and, in some cases, the skin may appear red-coloured due to the underlying inflammation. It often affects one or more digits asymmetrically and may be the first symptom of the SpA [41]. However, it is important to distinguish between dactylitis and non-dactylitic finger swelling, as the latter may not be associated with the same prognostic implications [39]. The Leeds Dactylitis Instrument (LDI) is a clinical tool that can help assess the risk of dactylitis in a swollen finger, based on the degree of swelling relative to the contralateral digit [42]. Dactylitis has been observed in 2–4% of IBD patients and is often related to the IBD disease activity [43]. Furthermore, individuals with CD exhibit a significative higher predisposition to dactylitis compared to those with UC, and this risk is correlated with elevated intestinal disease activity [44].

### 4.2. Minor Criteria

#### 4.2.1. Musculoskeletal Chest Pain

Musculoskeletal chest pain refers to discomfort or pain in the chest region that originates from joint structures, such as the costovertebral joints, costal cartilage, and sternoclavicular joints [26]. Musculoskeletal chest pain is one of the first signs of inflammation in the axial skeleton, especially in the costovertebral and costosternal joints, and may precede the development of more typical signs of SpA [45]. For that reason, chest pain in IBD patients, particularly when associated with other musculoskeletal symptoms (e.g., back pain, joint swelling), should raise the suspicion for inflammatory joint involvement [46].

#### 4.2.2. Family History for SpA

In addition, a positive family history for SpA could serve as a further indicator for clinicians to consider when evaluating patients with IBD who could show suggestive features of musculoskeletal involvement [47]. Recently, a large cohort study of the Swedish population revealed a 40% increased risk of SpA in first-degree relatives of patients with IBD; however, it even showed a 26% increased risk in spouses of IBD patients, suggesting other risk factors related to shared lifestyles [48]. Although family history alone is not definitive, it is one of the factors that should be considered in the overall clinical assessment, alongside other mentioned red flags. In fact, when a patient with IBD has a family history of SpA, it increases the suspicion of an associated musculoskeletal involvement and suggests the importance of further investigation and potential referral to a rheumatologist for confirmation and management.

#### 4.2.3. Psoriasis

Psoriasis has been described in patients with IBDs, and it is included as a minor criterion for suspicion of IBD-SpA. In fact, the presence of skin lesions attributable to classic psoriasis or other forms, particularly on the nails or scalp, or inverse psoriasis, may be considered to be a higher risk for developing SpA [49]. Despite the prevalence of IBD in patients with psoriasis being 3%, a meta-analysis revealed that patients with psoriasis had a 2.53-fold and 1.71-fold increased risk to develop CD and UC, respectively [50]. The presence of psoriasis in an IBD patient should raise suspicion for the overlap with psoriatic arthritis, even if the patient does not show the typical joint involvement yet [50]. However, the causative effect of both psoriasis and psoriatic arthritis on IBD manifestation still lacks evidence [51]. Patients with IBD may have a risk of developing psoriatic arthritis that is up to twice as high as the general population [52]. In addition, psoriasis in IBD patients may either precede, coincide with, or follow the onset of gastrointestinal symptoms. If psoriasis appears before the gastrointestinal symptoms, it may be an early indicator of an underlying SpA [51].

#### 4.2.4. Anterior Uveitis

Anterior uveitis is a well-known extra-intestinal manifestation of IBD-SpA characterized by ocular symptoms such as eye pain, redness, and blurred vision [53]. The presence of anterior uveitis in these patients may be an important sign that musculoskeletal involvement could be simultaneously present or about to develop [54]. In fact, anterior uveitis may indicate that the inflammatory process is not limited to the gastrointestinal tract but is affecting other tissues, like joints. A prompt treatment of this condition is mandatory to prevent important complications [55].

## 5. Screening Tools

In recent years, several questionnaires have been developed to identify IBD-SpA [56,57,58,59]. The Toronto Axial Spondyloarthritis Questionnaire for IBD (TASQ-IBD) is a diagnostic tool designed to identify SpA in patients with IBD, particularly those at higher risk of developing SpA [56]. It targets patients who experience chronic lower back pain or morning stiffness lasting at least three months. The questionnaire includes 16 items and a spine diagram, with a focus on the axial forms of SpA, which affect the spine and sacroiliac joints [56]. A reliability study involving 77 patients diagnosed with both SpA and IBD found that TASQ-IBD demonstrated high test–retest reliability, supporting its clinical usefulness. However, the tool primarily addresses axial SpA and may be less effective for detecting peripheral joint disease, which affects the limbs and other joints outside the spine. In summary, while TASQ-IBD is a valuable tool for diagnosing axial SpA in IBD patients and reducing diagnostic delays, its effectiveness may be limited in cases involving primarily peripheral joint involvement [56].

The DETection of Arthritis in Inflammatory Bowel Diseases (DETAIL) is another self-administered, validated questionnaire composed of six dichotomous clinical items. This tool is designed to quickly identify patients with IBD who exhibit joint signs and symptoms indicative of SpA [57]. In a cross-sectional study of 128 patients with CD or UC, the sensitivity of the DETAIL questionnaire ranged from 0.43 to 0.76 per item, while specificity varied from 0.62 to 0.89 per item. A post-test probability of ≥75% or a score of ≥3 positive answers was strongly suggestive of underlying SpA. However, the items in DETAIL were not selected based on psychometric analysis, and the questionnaire primarily focuses on peripheral joint involvement, including hand or foot arthritis, dactylitis, and Achilles enthesitis [57]. Although DETAIL serves as a useful screening tool for SpA in IBD patients, its scope is limited in terms of the peripheral joint conditions it assesses [57]. In addition, the IBD Identification of Spondyloarthritis Questionnaire (IBIS-Q) has been recently proposed as a more detailed screening tool, although it still requires further validation [58]. The Italian version of the IBIS-Q includes 42 items, 14 of which were chosen based on psychometric analysis. Unlike the DETAIL questionnaire, the IBIS-Q covers a wider range of arthropathies and enthesopathies, focusing on how these conditions affect a patient’s quality of life. A recent study comparing the performance of the DETAIL and IBIS-Q questionnaires in screening for IBD-related arthritis in 203 patients found that DETAIL had higher specificity but lower sensitivity compared to IBIS-Q. Specifically, DETAIL had a sensitivity of 40.0% (12.2–73.8) and a specificity of 84.4% (78.0–89.6), while the IBIS-Q showed a sensitivity of 70.0% (34.8–93.3) and a specificity of 74.3% (66.9–80.7) [59].

## 6. Laboratory Findings

Although several biomarkers have been proposed, laboratory findings still primarily support the diagnosis. C-reactive protein (CRP) is widely regarded as a key marker of disease activity in various inflammatory conditions [58]. In IBD patients with IBP, an elevated CRP may serve as a further laboratory red flag, suggesting the need for a rheumatologic referral [59]. Although CRP levels are commonly elevated during active gastrointestinal inflammation in IBD, sustained or raising CRP levels despite apparent control of intestinal symptoms may suggest extra-intestinal involvement, like the development of SpA [60]. However, CRP lacks both sensitivity and specificity for diagnosing SpA, and its levels may not be significantly different between IBD patients with or without SpA [61]. Thus, CRP levels should be carefully contextualized in the clinical picture of patients. Tissue typing for Human Leukocyte Antigens (HLAs) loci B and C is an additional laboratory test employed to further characterize patients with SpA associated with IBD [62]. In fact, those specific loci have been recognized as genetic risk factors for the development of musculoskeletal involvement in IBD patients [63]. Since the HLA loci B and C have been implicated in various SpA, their diagnostic utility has been subject to recent revaluation [64]. In contrast to other spondyloarthropathies, IBD is primarily associated with genetic variations in the promoter region of the tumor necrosis factor (TNF) gene. The most significant association is with HLA-B27, which has been found to carry a 10–40% risk of developing SpA features in IBD patients. However, HLA-B27 is common in the general population, and only around 5% of individuals who are positive for HLA-B27 will develop clinical SpA [65]. Additionally, Type I peripheral arthritis has been linked to HLA-B27 and HLA-B35, while Type II arthritis is more strongly associated with HLA-B44 and the MICA*008 variant of the Major Histocompatibility Complex Class I-related protein A (MICA) [66]. This highlights that while HLA typing may offer useful insights, in cases of IBD-related SpA, its clinical value is complex and depends on the specific genetic markers and their associations with different forms of arthritis. Nevertheless, the absence of a reliably accurate biomarker for IBD-SpA diagnosis necessitates a continued reliance on clinical correlation [67].

## 7. Imaging

As already mentioned, enthesitis, tenosynovitis, and dactylitis are frequently observed in patients with IBD [25]. Enthesitis is often overlooked during clinical examinations but may be detected earlier with ultrasound imaging of the affected regions. Despite pain being highly suggestive of clinical enthesitis, a musculoskeletal ultrasound with B mode power Doppler should be performed as a first line to identify tendon edema, peri-tendinitis, enthesis erosion, and adjacent bone marrow edema [68]. While tenderness at enthesis sites may be a salient clinical finding in approximately one-third of IBD patients, chronic enthesopathy, as evidenced by ultrasonographic studies, has been documented in up to 88.2% of cases, particularly among those with a protracted disease course [69]. In addition, X-rays may show evidence of enthesophytes or bone erosions in chronic cases. Finally, MRI is highly sensitive for detecting early signs of inflammation in the entheses, particularly in the spine and pelvis, and may reveal subtle changes before they appear on X-ray [70]. In patients with IBD experiencing lower back pain, an anteroposterior pelvic radiograph is recommended to assess for sacroiliitis [71]. However, the variation in frequencies about inflammatory spine involvement may be attributed to differences in diagnostic methods, which could be based on clinical symptoms, plain radiographs, or cross-sectional computed tomography images. These different methods of reporting the IBP and spine involvement could suggest the need for further studies to achieve standardization [72]. MRI, particularly with the short-tau inversion recovery technique, is highly effective in detecting sacroiliitis and enthesitis, revealing inflammation, bone marrow edema, and bony erosions that are not visible on conventional radiographs [73]. Notably, similar findings may often be obtained from magnetic resonance enterorrhaphy in these patients; thus, it is advisable to inform the radiologist interpreting the magnetic resonance enterorrhaphy to examine the bones and joints for such complications [74]. The diagnosis of dactylitis is mainly clinical. However, further diagnostic steps may be necessary [75]. In particular, ultrasound scans or X-rays of the affected finger may be useful to discriminate this clinical condition from other causes of joint swelling [75].

## 8. Treatment

**Figure 3:**
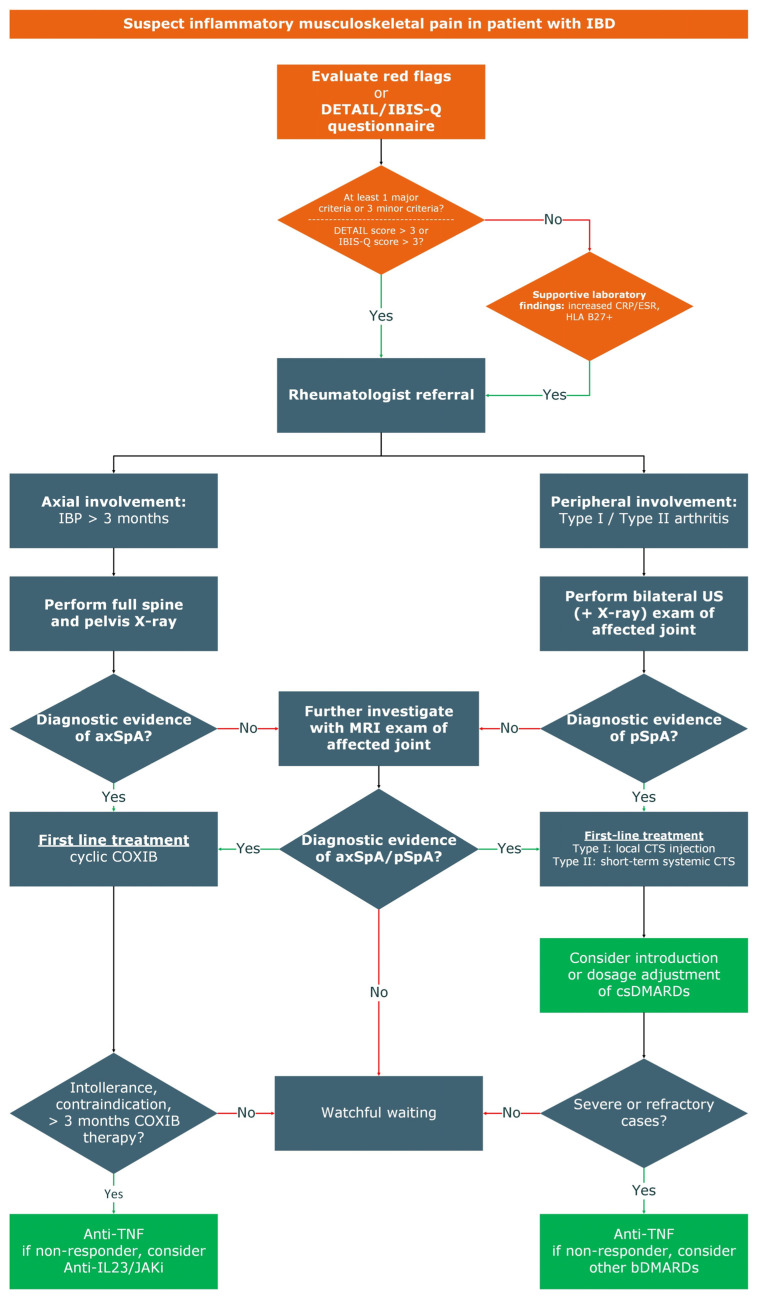
Proposed diagnostic and therapeutic management of patients with IBD and musculoskeletal inflammatory involvement.

When IBD and SpA coexist, musculoskeletal and inflammatory intestinal features should be considered when planning a therapeutic strategy to be simultaneously targeted. In treating a patient with SpA occurring simultaneously with IBD, it is appropriate to give priority to the active disease. Collaborative care between rheumatologists and gastroenterologists is thus essential for a personalized approach to patient management. Recently, the Italian Group for the Study of Inflammatory Bowel Disease (IG-IBD) and Italian Society of Rheumatology (SIR) proposed recommendations for the management of SpA-related arthritis [76]. These recommendations consider the disease activity of IBD and musculoskeletal involvement together with the presence of either an axial or peripheral joint pattern [71]. The authors proposed 34 statements to manage these patients and practical therapeutic algorithms to help clinicians in their daily clinical practice. As stated in EULAR’s last recommendations, NSAIDs are considered a first-line drug treatment in patients with IBP [77]. IBP pain responsive to 2–4 months of treatment with NSAIDs could be considered as an additional red flag of SpA in IBD patients. However, these drugs should be carefully administered in IBD patients due to the risk of gastrointestinal side effects. In this context, COX-2 inhibitors (COXIB) could be more suitable for IBD patients [78]. Moreover, COXIB benefits could be partial and should not be considered as long-term treatment [73]. Considering its well-proven efficacy in both conditions, anti-TNF therapy remains the cornerstone in the treatment of these patients [79]. Other therapeutic options such as Janus kinases (JAK) inhibitors and interleukin (IL)-23 inhibitors, are considered in non-responder patients or in case of intolerance to first-line therapies [80,81]. In peripheral SpA, local glucocorticoid injections could be considered, especially in Type I arthritis and inactive IBD. Instead, long-term steroid therapy should be avoided and only considered as bridge therapy [82]. Type II arthritis or refractory Type I arthritis should be further investigated for the potential introduction or dosage adjustment of a background conventional synthetic disease-modifying antirheumatic drugs (csDMARDs) (sulphasalazine in UC, methotrexate in CD) treatment [83]. The treatment of dactylitis involves addressing the underlying inflammatory condition and may include the use of NSAIDs or, in case of more severe or refractory cases, biological DMARDs (bDMARDs) may be considered [79]. A recent retrospective cohort study of 507 IBD patients evaluated the impact of different treatment strategies on SpA manifestations. The study demonstrated that bDMARDs therapy may effectively prevent the development of SpA in patients with CD, both in the short and long term [84]. Delayed diagnosis of SpA in IBD patients has significant consequences for patient outcomes. With a global average diagnostic delay of 6.7 years for IBD-associated SpA, this delay is considered unacceptable, given the high risk of chronic complications [85]. Additionally, starting appropriate treatments, such as anti-TNF agents, early in the disease can improve patient outcomes. The collaboration between gastroenterology and rheumatology in specialized settings is important for the early detection of SpA in IBD patients [86,87,88]. This integrated approach facilitates a thorough assessment of both gastrointestinal and musculoskeletal symptoms, leading to earlier identification of patients at risk for SpA [89]. In Figure 3 we summarize a diagnostic and therapeutic management of these patients.

## 9. Conclusions

A multidisciplinary approach involving both gastroenterologists and rheumatologists is essential for improving the management of patients IBD-SpA. Due to the overlap of these conditions, it is crucial for both specialists to collaborate in developing a comprehensive treatment plan. This ensures that both the intestinal and musculoskeletal aspects of the disease are addressed simultaneously, allowing for a tailored treatment strategy based on the patient’s unique clinical profile. In this context, recognizing red flags plays a critical role in optimizing clinical, diagnostic, and therapeutic management. Delayed diagnosis and treatment are common in these patients due to the complex nature of their symptoms. Therefore, early intervention is crucial to achieve remission and improve long-term patient outcomes.

## Data Availability

Not applicable.
